# A Rare Case of Ileo-Ileal Knotting: A Case Report

**DOI:** 10.7759/cureus.41903

**Published:** 2023-07-14

**Authors:** Wongel Tena Shale, Langa James Oriho

**Affiliations:** 1 College of Public Health and Medical Sciences, Department of Surgery, Jimma University, Jimma, ETH

**Keywords:** compound volvulus, acute abdomen, gangrenous small bowel, intestinal obstruction, intestinal knotting, ileo-ileal knotting

## Abstract

Small bowel obstruction due to ileo-ileal knotting is rare. Ileo-ileal knotting usually presents with clinical features of small bowel obstruction with rapid deterioration to bowel necrosis, and the management includes prompt surgical intervention. Here, we present a case of a 35-year-old patient who presented to our emergency room with severe non-radiating crampy abdominal pain for 18 hours. The patient underwent an emergency laparotomy as an intervention. Ileo-ileal knotting preoperative diagnosis is challenging due to its nonspecific presentation, the diagnosis is usually done intraoperatively, and the overall management of gangrenous ileo-ileal knotting is urgent laparotomy and en bloc resection of the gangrenous ileo-ileal knotting and anastomosis of the remaining viable bowel. If the bowel is viable, careful untying of the loops usually suffices. Ileo-ileal knotting should be considered in patients presenting with features of small bowel obstruction having rapid deterioration with signs of gangrenous bowel, and it requires urgent surgical intervention after adequate resuscitation.

## Introduction

Intestinal knot formation occurs when two intestinal segments intertwine together to form a knot or compound volvulus, resulting in intestinal obstruction and impaired blood flow [1]. Most intestinal knot formation is found between the sigmoid colon and ileum, the so-called "ileosigmoid knotting," whereas knot formation between two segments of the ileum is rare, with only a handful of cases reported in the literature [1-13]. A study by Shepherd et al. (1967) [2] stated that ileo-ileal knotting was found in only one of 92 cases of intestinal knots. The etiology of ileo-ileal knotting is not known, and risk factors suggested include mobile small bowel with long mesentery, sudden or vigorous bowel movement, single bulky meal, and pregnancy [8,13,14]. Ileo-ileal knotting usually presents with clinical features of small bowel obstruction with rapid deterioration to bowel necrosis, and preoperative investigations are usually nonspecific [8]. A high clinical index of suspiciousness is required to diagnose ileo-ileal knotting [6]. Here, we present a case of a 35-year-old male patient who presented with ileo-ileal knotting.

## Case presentation

A 35-year-old male patient presented to our emergency room with severe non-radiating crampy abdominal pain for 18 hours. Associated with this, he had frequent vomiting initially consisting of ingested matter and later became bilious, and he also failed to pass feces since the presentation, but he could pass flatus. He had a history of long-standing dyspepsia and was on proton pump inhibitors. There was no significant history of similar conditions or previous surgery. He denied any history of alcohol consumption or tobacco use. His regular diet was injera, which is a fermented Ethiopian traditional staple food prepared from teff flour. Upon arrival, he was in pain, and his vital signs were as follows: blood pressure of 97/66 millimeter of mercury (mmHg), pulse rate of 130 beats/minute (bpm), respiratory rate of 24 breaths/minute, temperature of 37.1°C, and oxygen saturation of 96% with atmospheric air. His conjunctivae were pink, and his tongue and buccal mucosa were dry. The abdomen was mildly distended and moved with respiration, bowel sounds were active, and there was tenderness all over on deep palpation, but no guarding or rigidity. A digital rectal examination showed an empty rectum with no blood or mass. With an impression of gangrenous small bowel secondary to small bowel obstruction, he was resuscitated with 2 L of normal saline within one hour. His blood pressure increased to 110/80 mmHg, his pulse rate was 96 bpm, and he produced 100 milliliters (mL) of urine. He had a nasogastric (NG) tube output of 1 L/one hour of bilious content after insertion.

**Figure 1:**
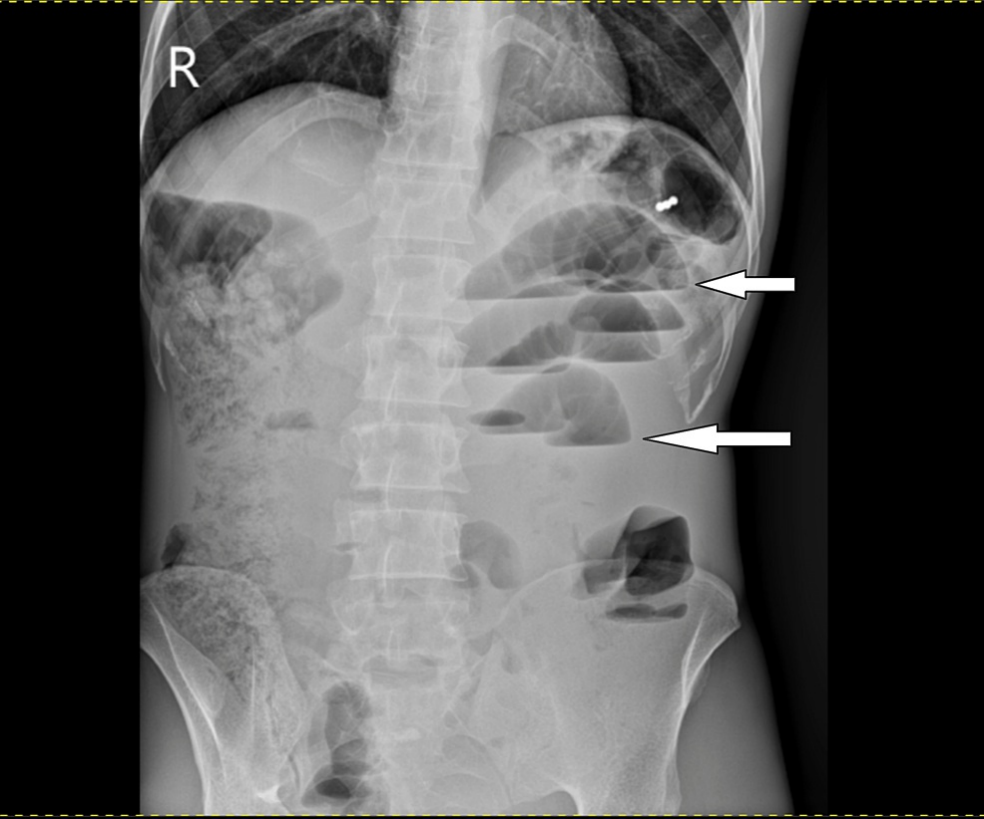
Multiple centrally located air-fluid levels and distended bowel loops (white arrows) depicting small bowel obstruction

He was investigated with complete blood count (CBC): white blood cell count (WBC) of 15,360/microliter (μL) (normal range: 3,000-15,000/μL), 89.6% neutrophils (normal range: 37%-72%), hemoglobin of 12.2 gram/deciliter (g/dL) (normal range: 8-17 g/dL), 34.9% hematocrit (normal range: 25%-50%), and platelets of 406,000/μL (normal range: 150,000-450,000/μL). An erect plain abdominal X-ray was taken and showed a dilated small bowel with multiple air-fluid levels, as can be seen in Figure 1.

**Figure 2:**
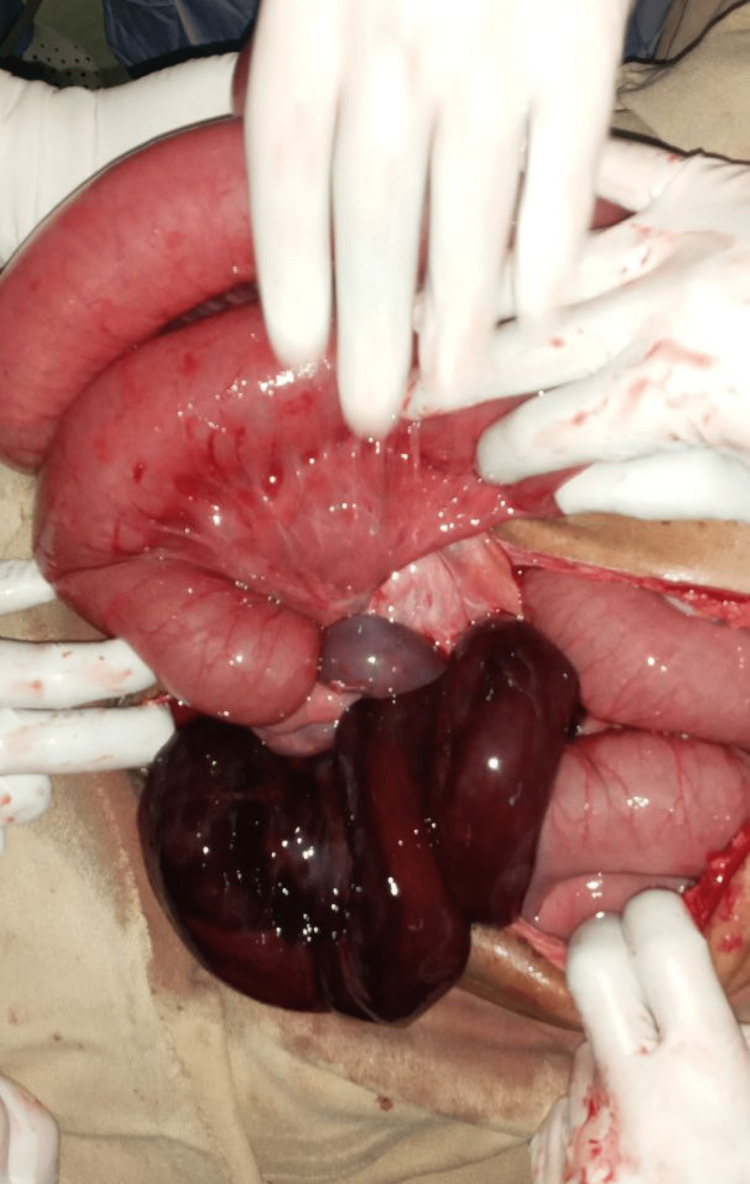
Intraoperative image of an ileo-ileal knotting with gangrenous ileum

Antibiotics were given, ceftriaxone and metronidazole, and he was taken to the operation room for urgent exploratory laparotomy after consent was taken. A midline laparotomy incision was made, and there was 300 mL of hemorrhagic fluid upon entering the peritoneum. The proximal loop of the ileum was knotting on the distal ileum. The entrapped loop of the ileum was gangrenous, extending up to 7 cm from the ileocecal valve as can be seen in Figure 2. It was difficult to clamp and ligate the mesentery en bloc, which could endanger the blood supply of the viable segment. A controlled enterotomy was done to untie the knot, and mesenteric vessels were ligated. Resection of the 100 cm of gangrenous ileum was done, and end-to-end ileo-ileal anastomosis was performed to restore gut continuity. The proximal small bowel measuring 280 cm and distal ileum from the ileocecal valve measuring 7 cm remained.

The patient experienced paralytic ileus and moderate anemia (8.4 g/dL) after surgery. Hypoalbuminemia (2.27 milligrams/deciliter) and a superficial surgical site infection also complicated his recovery time. He received therapeutic oral iron replacement therapy, a diet high in protein, and wound care. On the 12th POD, he was released from the hospital after completing his recovery.

## Discussion

Intestinal knotting was first described in the 16th century by Riverius, and Rokitansky further described this unusual condition in detail in 1836. Ileosigmoid and ileo-ileal knotting are extremely uncommon conditions that can quickly progress to gangrene of the afflicted bowel segments, especially in Western nations [4]. It is a relatively uncommon cause of acute intestinal obstruction and bowel gangrene and involves the twisting of the bowel loops with an intervening knot. To form a knot, a dynamic ileal loop must twist and loop around a relatively static ileal coil. Ileosigmoid knotting is the most prevalent type of intestinal knotting [14-17]. There have only been a few isolated occurrences of ileo-ileal knotting reported worldwide [1-13]. A systematic search was conducted for literature published up to December 2019 using different databases. There were 13 instances of ileo-ileal knotting on all platforms combined. There have been cases documented in people aged 11 months to 80 years old, and neither age nor sex seems to be a clear factor. Reports of the entity have come from Africa, Asia, and Eastern Europe, but for some reason, it is less common in the West [8]. The cause of intestinal knotting, particularly ileo-ileal knotting, is unknown [8,18]. Where primary sigmoid and small intestine volvulus are frequent, the disease is most common. A bulky, high-fiber diet may be responsible for this [8,14,15]. After an extensive search of the literature, a total of five cases of ileo-ileal knotting were reported from Ethiopia (Table 1).

**Table 1:** Reported cases of ileo-ileal knotting in Ethiopia ICV: ileocecal valve

Author	Age (years)	Sex	Chief complaints	Location of knot	Bowel viability	Type of surgery	Outcome	Hospital length of stay (days)
Abebe et al. (2015) [12]	55	Female	Abdominal pain, vomiting, abdominal distention	8 cm from the ICV	Gangrenous	Resection of the gangrenous segment and end-to-end jejunoileal anastomosis	Short bowel syndrome, discharged	14
Mohammed et al. (2021) [10]	18	Female	Abdominal pain, vomiting, diarrhea	3 cm from the ICV	Gangrenous	Resection of the gangrenous segment, right hemicolectomy, and ileotransverse anastomosis	Discharged	6
Knfe et al. 2023 [7]	13	Male	Abdominal pain, abdominal distension, nausea, vomiting	30 cm from the ICV	Gangrenous	Resection of the gangrenous segment and end-to-end ileo-ileal anastomosis	Discharged	6
Knfe et al. 2023 [7]	12	Female	Abdominal pain, fever, vomiting		Gangrenous	Resection of the gangrenous segment and end-to-end ileo-ascending anastomosis	Discharged	7
Mohammed et al. (2023) [13]	35	Female	Abdominal pain, vomiting, abdominal distension	10 cm from the ICV	Viable	Untying	Discharged	5
Tena Shale et al. (2023) (this study)	35	Male	Abdominal pain, vomiting, failure to pass feces	7 cm from the ICV	Gangrenous	Resection of the gangrenous segment and end-to-end ileo-ileal anastomosis	Moderate anemia, hypoalbuminemia, paralytic ileus, improved and discharged	12

Ileo-ileal knotting has a similar presentation to small bowel obstruction from any other cause; however, individuals with ileo-ileal knotting typically become worse quickly. The symptom that is most frequently stated is abdominal pain. The patient is awakened from sleep by the sudden onset of pain, which most frequently happens in the early morning hours. Vomiting is also prevalent and usually happens simultaneously with pain. Abdominal distention is not a predominant characteristic of ileo-ileal knotting, in contrast to primary volvulus. The condition typically advances quickly to intestinal ischemia, gangrene, and perforation. As a result, patients display symptoms of peritonism [13]. According to the systematic review by Beg et al. [8], the most frequent presenting complaints were abdominal pain (93%), vomiting (64%), abdominal distention (57%), and constipation (43%), with an average time to present to the hospital of two days. Diagnosis of ileo-ileal knotting is mostly done intraoperatively [10]. This was also reflected in the way the case presented above. He typically displayed significant, ongoing pain in the abdomen, vomiting, and mild abdominal distention. He had severe tachycardia and borderline blood pressure, both signs of hypovolemia. Evidence suggests that the mortality rate may be as high as 50% [4].

It is challenging to diagnose intestinal knotting before surgery. It is possible to consider coexisting illnesses or more frequent causes of intestinal obstruction [10]. Almost always, the diagnosis is made during surgery. A plain erect X-ray of the abdomen will demonstrate the features of intestinal obstruction. If necessary, a computed tomography (CT) scan of the pelvis and abdomen might be performed [19]. On an abdominal CT scan, signs of closed-loop obstruction could be visible. A volvulus may be indicated by abdominal CT scan findings, including the recognizable "whirlpool sign." A CT scan can also detect mesenteric edema and other symptoms of bowel ischemia brought on by strangulation, such as pneumatosis intestinalis, thickening of the gut wall, and peri-colic fluid collection [5,20].

Treatment should begin as soon as feasible with rapid intravenous (IV) fluid resuscitation, placement of a nasogastric tube, and broad-spectrum IV antibiotics. Treatment should not be delayed owing to investigating patients. After the patient has undergone adequate resuscitation, a high index of suspicion is required, and quick abdominal exploration is warranted. If the intestine loops are still viable, intestinal knots can be carefully untied in cases of knotting. When gangrenous bowel is discovered, an en bloc resection of the affected segments is the recommended approach. It is not advised to decompress gangrenous segments or untie gangrenous knots. Despite the fact that en bloc resection can be difficult, there is a potential for these maneuvers to raise the risk of reperfusion injury. It also poses a very significant risk of peritoneal contamination from the spillage of contents of the gangrenous bowel [4,10-12]. The patient's general condition will determine whether or not it is necessary to undergo primary anastomosis to restore gastrointestinal continuity. Most individuals who have stable hemodynamics have primary anastomosis. A stoma is used to manage some patients who are persistently in shock or have severely edematous intestines. It was challenging to clamp and ligate the mesentery in our situation. Despite the fact that it is not ideal, a controlled enterotomy was performed to untie the knot, and mesenteric arteries were quickly ligated with the removal of the gangrenous segment. In hindsight, ligation of the mesenteric arteries and piecemeal resection of the gangrenous bowel could have been tried instead of enterotomy and untying the gangrenous segment. Fortunately, there was no enteric contamination of the peritoneum, and the patient did not experience intra-abdominal collection during the course of recovery.

If an anastomosis is performed, the patient should have postoperative monitoring for signs of anastomotic leak, anemia, and hydration status. If a stoma is developed, it needs to be cared for. The patient should receive advice on how to care for their stoma and when it will be closed. Our patient did not develop an anastomotic leak or intra-abdominal abscess during the postoperative course.

Regardless of its rare occurrence and challenges in the diagnosis, ileo-ileal knotting should be suspected in any bowel obstruction with rapid clinical progression and deterioration, and prompt surgical intervention is highly required.

## Conclusions

Ileo-ileal knotting should be considered in patients presenting with features of small bowel obstruction having rapid deterioration with signs of gangrenous bowel, and it requires urgent surgical intervention after adequate resuscitation.
